# Phonological Feature Abstraction Before 6 Months: Amodal Recognition of Place of Articulation Across Multiple Consonants

**DOI:** 10.1111/desc.13605

**Published:** 2025-01-14

**Authors:** Eylem Altuntas, Catherine T. Best, Marina Kalashnikova, Antonia Götz, Denis Burnham

**Affiliations:** ^1^ MARCS Institute for Brain, Behaviour, and Development Western Sydney University Sydney Australia; ^2^ Basque Center on Cognition Brain, and Language Donostia/San Sebastián Spain; ^3^ Ikerbasque Basque Foundation for Science Bilbao Spain

**Keywords:** amodal speech perception, artificial language learning, early infancy, infant speech perception, perceptual attunement, phonological feature abstraction

## Abstract

The classical view is that perceptual attunement to the native language, which emerges by 6–10 months, developmentally precedes phonological feature abstraction abilities. That assumption is challenged by findings from adults adopted into a new language environment at 3–5 months that imply they had already formed phonological feature abstractions about their birth language prior to 6 months. As phonological feature abstraction had not been directly tested in infants, we examined 4–6‐month‐olds’ amodal abstraction of the labial versus coronal place of articulation distinction between consonants. In the training phase, infants heard a series of labial non‐words paired with an animal image and a series of coronal non‐words (multisyllabic) paired with another image. At test, they viewed a silent video of a talker producing coronal and labial words, paired with either the familiarised image or the contrary image. The infants looked significantly longer on matching trials than mismatching trials, suggesting amodal abstraction of this consonantal place of articulation distinction by 4–6 months. These findings provide direct evidence for the inference from the adoptee findings that phonological feature abstraction emerges prior to perceptual attunement.

## Introduction

Infants rapidly learn the speech patterns of their native language during their first year, acquiring essential phonetic properties unique to that language in a process called perceptual attunement. This process involves maintenance of initially good universal discrimination, or even increased sensitivity, for native consonant and vowel contrasts along with diminished sensitivity to non‐native ones, those not found in infants’ native language, during the second half of the first year of life. In essence, infants become more attuned to the specific speech segments (consonants and vowels) relevant to their linguistic environment. Numerous studies provide evidence for this perceptual tuning, particularly the decline in discrimination of most non‐native contrasts (Best 1993; Kuhl et al. 2006; Werker 1989). The emergence of perceptual attunement differs for vowels and consonants (Cs). Around 6 months of age, infants begin to exhibit native vowel attunement, demonstrating a decrease in their ability to discriminate non‐native vowel contrasts (Kuhl et al. 2006; Polka and Werker 1994). For consonant attunement, the decline in discrimination for non‐native contrasts typically is evident by around 10 months of age (Tsuji and Cristia 2014; Werker and Tees 1984). It is important to note, however, that there are certain systematic exceptions to these perceptual patterns for both native (Polka, Colantonio, and Sundara 2001; Sundara, Polka, and Genesee 2006) and non‐native contrasts (Best et al. 1995; Best, McRoberts, and Sithole 1988; Polka and Bohn 1996; for a critical review, see Best et al. 2016). For instance, English‐learning infants show good perceptual differentiation of the native [d—ð] contrast only after reaching 12 months of age (Polka, Colantonio, and Sundara 2001), while 12–14‐month‐old English‐learning infants continue to discriminate non‐native Zulu clicks (Best et al. 1995). These latter ‘exceptions’ are argued to reflect infants’ sensitivity to variations in articulatory properties in speech (Best et al. 2016), an idea central to the study reported here.

**Summary**

- Infants younger than 6 months show encoding of articulatory classes by distinguishing mini‐languages by consonant place of articulation (labial vs. coronal).
- Cross‐modal design shows infants generalise abstract phonological features from audio training to testing with silent talking video.
- Findings align with the Perceptual Assimilation Model (PAM) framework, highlighting amodal articulatory information as a basis for infants’ phonological feature abstraction.
- Results challenge current understanding by showing phonological abstraction prior to perceptual attunement.

Perceptual attunement is generally regarded as the foundation for subsequent development of more abstract processes, such as those involved in recognising native words and making phonological feature abstractions about the native language. Thus, the assumption has been that phonological feature abstraction follows, rather than precedes, the emergence of perceptual attunement to the native language (Jusczyk 1993; Werker and Curtin 2005; Werker and Tees 1984). Jusczyk's (1993) Word Recognition and Phonetic Structure Acquisition model (WRAPSA) proposes that infants’ early speech perception abilities rely on general (not specific to speech), universal (cross‐language) auditory analysers. However, as infants become familiar with their native language's phonetic structure, they start weighing information from those analysers in order to develop phonetic categories for native consonants and vowels, from which they then begin to compute more abstract phonological features and contrasts that are specific to that language. Thus, WRAPSA assumes that perceptual attunement precedes phonological feature abstraction in infants’ speech perception development.

Some researchers propose that phonological feature abstractions develop even later, arguing that infants must first acquire a sufficiently large repertoire of words they know (recognise) before engaging in phonological feature abstraction (Beckman and Edwards 2000). The Processing Rich Information from Multidimensional Interactive Representations (PRIMIR) framework also posits that phonological feature abstractions emerge after the onset of word learning (Werker and Curtin 2005). According to PRIMIR, infants initially focus on perceptual details and create representations for specific words. Once they have a sufficient number of words they recognise, infants can then generalise and abstract underlying phonological patterns, forming more abstract consonant and vowel representations (phonemes). Additionally, the lexical restructuring hypothesis (Walley 1993) suggests that during infancy and early childhood, children's lexical representations and processing are initially holistic and non‐segmental. However, as they gain vocabulary, they start to decipher their native language's segmental structure and gradually shift toward restructuring their lexical representations. This restructuring coincides with the vocabulary growth spurt around 18 months, as it reflects the need for more detailed and efficient storage of lexical items, organised by their phonemic subcomponents, that is, consonants and vowels. Thus, vocabulary growth contributes to the development of phonological representations.

Those views posit that perceptual attunement, or lexical development itself, serves as a foundation for subsequent phonological feature abstraction. The assumption is that as infants refine their perception of native speech segments and phonemic components of words, this in turn leads to the emergence of more abstract phonological representations. These theoretical assumptions underscore the presumed necessity of perceptual attunement as a precursor to engaging in phonological feature abstraction.

However, two recent studies challenge the traditional belief that perceptual attunement must precede and pave the way for phonological feature abstraction (Choi, Broersma, and Cutler 2017; Choi, Cutler and Broersma 2017). Their findings suggest that phonological feature abstraction may precede perceptual attunement, occurring even before 6 months. In Choi, Broersma, and Cutler (2017), 30‐year‐old Dutch speakers adopted from Korea at 3 to 5 months of age, who had no subsequent exposure to the Korean language, exhibited remarkable abilities for learning Korean‐only consonant contrasts. They quickly learned to identify the Korean 3‐way fortis, lenis, and aspirated alveolar stop contrast ([t*]‐[t]‐[t^h^]), which does not exist in Dutch, outperforming their Dutch counterparts who lacked prior Korean exposure. Furthermore, these adoptees excelled in generalising the identification of this 3‐way contrast from the trained alveolar place of articulation to bilabial and velar places without any additional training. In addition, only they successfully produced the contrast at all three places, more accurately generalising from perception to production than the Dutch controls (Choi, Broersma, and Cutler 2017). These findings present a conflict for the conventional wisdom that perceptual attunement precedes phonological feature abstraction. The adoptees' exceptional performance in discriminating and generalising these Korean‐only phonemic contrasts across articulatory contexts, despite not experiencing Korean since 3–5 months of age, suggests that abstract phonological knowledge had developed prior to native language perceptual attunement at 6–10 months. While Choi and colleagues' studies offer compelling indirect evidence for early phonological feature abstraction before perceptual attunement develops, there is to date no direct evidence of phonological feature abstraction in infants younger than 6 months.

As phonological feature abstraction is crucial for language acquisition and literacy, it is important to understand when and how infants begin to employ this skill. Infants *older* than 6 months display a range of linguistic abstraction abilities, including phonological feature abstraction, which we illustrate here with two key studies. One found that 7‐month‐olds can identify and generalise permissible word onsets in artificial languages defined by the phonological feature of manner of articulation (Cristia and Seidl 2008). Infants were trained on a set of nonsense words, half of which started with nasals (e.g., *noog* [nug], *moz* [maz]) while the other half started with stops for one group (e.g., *gawp* [gɔp], *teel* [til]), or with fricatives for the other group (e.g., *fawng* [fɔŋ], *zeed* [zid]). They were then tested with new words, half of which started with new stops (e.g., *boj* [badʒ], *keet* [kit]) and half with new fricatives (e.g., *shof* [ʃaf], *veech* [vitʃ]). Despite the novelty of all onset consonants during testing, the infants attended more to the untrained than the trained manner for their group, suggesting surprise at the unexpected consonant feature, which indicates that both groups had learned and generalised their respective phonological feature rule.

In the other study, 11‐month‐olds showed rapid phonological feature abstraction from sets of nonsense words in which the consonants (stops and fricatives) within each word shared a phonological feature (either same voicing, e.g., *pota* [potɐ], *beeza* [bizɐ]; or same place of articulation, e.g., *poba* [pobɐ], *teeza* [tizɐ]) (Gerken and Knight 2015). Infants in the non‐conflicting training groups exclusively heard words that either shared consonant voicing or shared place of articulation, while those in the partially conflicting groups heard words with either same‐voicing or same‐place (50%) and words with shared *consonants*, that is, same voicing, place, and manner (50%; e.g., *popa* [popɐ], *beeba* [bibɐ], *fofa* [fofɐ], *zeeza* [zizɐ]) within each word. The test phase presented new words displaying both types of shared phonological feature. The non‐conflicting groups showed a novelty preference for new words that violated their trained phonological feature, while the partially conflicting groups showed a familiarity preference for new words that conformed to their trained phonological feature. Thus, 11‐month‐olds learned both trained phonological feature rules even in the partially conflicting training condition, displaying impressive phonological feature abstraction abilities.

We know of only two studies that have provided limited evidence supporting the notion of phonological feature abstraction in infants *under* 6 months. However, these findings are confined to certain vowel and consonant contrasts. In the first, newborns and 2‐month‐olds were habituated to the syllable set [bi], [si], [li], [mi], then a new syllable was added to the set in the subsequent test phase (Bertoncini et al. 1988). There were five groups per age. One group at each age heard a new syllable that included the familiar vowel with a new consonant ([di]), while two other groups heard new syllables with the same new consonant but with a different new vowel ([du] or [da], respectively). Another group heard a new syllable with a new vowel but the same initial consonant as one of the familiarisation syllables ([ma]). The control group experienced no changes except for an increased frequency of occurrence of the familiarisation syllable [mi]. Responses were measured using the preferential sucking rate paradigm. The 2‐month‐olds successfully detected all additions introduced during the test phase, highlighting their ability to learn the phonological commonality of the habituation syllables (the shared vowel [i]) and to recognise that the newly added syllables contained new vowels and/or consonants. Conversely, the newborns apparently did learn that the habituation syllables all contained the vowel [i] but did not attend to the consonants, given that in the test phase they detected new syllables with new vowels but not those with new consonants. While hinting at early phonological feature abstraction, the authors suggested that this interpretation remains uncertain, because regardless of the vowel being novel or familiar, the addition of a new syllable consistently prompted a similar response. The authors interpreted the 2‐month‐olds’ responses as providing limited support for early speech sound representations organised around vowel segments. This interpretation rests on the assumption that, if infants organised their representations this way, a syllable with the same vowel as the familiarisation syllables should be less novel than one with a new vowel. The study thus offers limited endorsement for syllable representations based on segmental phonetic features in infants of this age, warranting further investigation into their capability for phonological feature abstraction.

The other study (Hillenbrand 1984) examined 6‐month‐olds’ ability to recognise that a set of syllables shared a common consonant, despite variations in phonetic context (vowels) and talkers. The experimental groups received training on a place of articulation contrast between labial and alveolar nasals ([ma], [na]). Initially, the training stimuli used a single male talker, and as the experiment progressed, novel tokens of the contrast were introduced, varying in vowel contexts (e.g., [mi], [mu], [ni], [nu]) and new talkers (both male and female). The control group received the same stimuli, but they were presented in a mixed order rather than paired as a contrast. Only the infants in the experimental group learned and successfully generalised the labial versus alveolar nasal contrast to novel tokens with different talkers and vowel contexts. Overall, the study demonstrated that 6‐month‐old infants can abstract a nasal place of articulation difference across variations in talkers and vowel contexts when it is initially presented as a contrast.

However, no studies to date have directly shown whether infants younger than 6 months can abstract and generalise phonological feature distinctions for consonant contrasts that are comparable to the findings of Choi, Broersma, and Cutler (2017) with adult adoptees. Thus, it is important to investigate whether and how phonological feature abstraction may take place in infants under 6 months, before perceptual attunement to native consonants (and even to native vowels) has been demonstrated. The current study investigated young infants’ abstraction of a native consonant feature contrast analogous to the one that Choi et al. examined in adult adoptees. To address this, we adapted a paradigm similar to Kabdebon and Dehaene‐Lambertz (2019), who showed that 5‐month‐old infants trained on nonsense words from two contrasting artificial mini‐languages differing only in syllabic structure learned the structural contrast and generalised it to new words. However, we modified the stimuli to suit our research objectives by distinguishing the mini‐languages’ words with respect to consonant place of articulation rather than syllabic structure.

We specifically chose a place of articulation contrast as our focus because this choice allowed us to investigate whether infants are capable of abstracting amodal articulatory information from speech across different modalities, specifically audio‐only and video‐only (silent talking face) presentations of spoken words. This decision was motivated by the Perceptual Assimilation Model (PAM) premise that the foundation of speech perception, perceptual attunement, and phonological feature abstraction lies in *amodal articulatory information* (Best 1993, 1994, 1995; Best et al. 1988, Best et al. 2016; Best and Tyler 2007), but it is also compatible with the cross‐modal perceptual narrowing account of infant perceptual attunement (e.g., Pons et al. 2009). For these reasons, we employed a cross‐modal transfer task for our experimental design, which involved testing cross‐modal generalisation of the abstraction of the place of articulation feature contrast by shifting from the training modality (audio) to another modality in the subsequent test phase (video).

Relatively little infant speech research has used cross‐modal transfer, particularly for investigations of phonological feature abstraction. The only two prior cross‐modal speech perception investigations both examined perceptual attunement to native versus non‐native consonant contrasts by comparing infants younger than 7 months and older than 10 months (Best et al. 2010; Pons et al. 2009). While these prior studies indicate 11‐month‐olds’ perceptual attunement to the native language affects their cross‐modal responses to non‐native consonant contrasts, the younger infants’ (6 or 4 months) responses suggest that they detect amodal place of articulation correspondence prior to perceptual attunement to consonants. However, we still do not know whether they had abstracted the phonological feature of place of articulation or simply detected the articulatory difference between the single consonant pair tested. Therefore, we examined whether infants under 6 months can abstract phonological feature information about labial (lips) versus coronal (tongue tip) across multiple consonants that share each place. Specifically, we assessed whether infants could learn to differentiate between two artificial languages that differ only in whether all of their consonants are produced by the lips or all are produced by the tongue tip, which would require abstraction of the phonological feature of place of articulation across multiple consonants in each language.

To investigate this phonological feature abstraction, we employed a cross‐modal transfer procedure with looking times as the dependent measure, training infants in one modality (audio‐only words) and testing their responses to new words of each language in a different modality (silent video‐only talking faces producing the words). We chose this direction of modality change based on our previous finding that audio training with video testing is optimal for infants to show cross‐modal transfer (Altuntas et al. 2023). Our mini‐languages included multiple consonants at each place of articulation to examine whether infants could learn and generalise the shared feature within each language, beyond simple specific consonant distinctions. The cross‐modal methodology enabled us to ascertain whether infants could distinguish between labial and coronal consonants based on amodal articulatory information, effectively matching the articulator used in the audio input during training with the articulator observed in the silent video during testing. If infants had learned the articulatory difference between the languages, they could, in principle, look longer to either the congruent or the incongruent test trials. We anticipated that infants would favour the congruent trials in our study, reflecting their familiarity with the word structure of each language combined with the shift in stimulus modality.

## 1 Method

### 1.1 Participants

The participants were 34 four‐ to six‐month‐old infants (M_age_ = 4.72 mo, SD = 0.56, range = 3.68–5.75). All infants were Australian English‐learning monolinguals in Sydney, Australia. All were Caucasian, born full‐term, with normal hearing and vision. Demographic data, such as infant's age, languages spoken at home and their percentages, and whether the infant was born full term and without any risk factors for cognitive or language impairment, were collected at the time of registration through a questionnaire. Further information was collected on the test day, such as family income, languages spoken at home in terms of hours in a week, and parental education. Annual family income ranged between less than $25,000 AU to more than $100,000 AU (median = $50,000–99,999 AU). Mothers’ educational levels ranged from not completing high school to achieving a master's degree (median = university bachelor's degree). We set the threshold for minimal exposure to languages other than English at no more than 4 h per week (M = 0.67, SD = 1.40, range = 0–4). Families were given a $20 AU travel reimbursement, a small gift, and a certificate of completion for their participation in the study.

### 1.2 Stimuli

The two mini‐languages were generated, each composed of multi‐syllabic nonsense words in which the syllable‐initial consonants all shared the same place of articulation. The words of mini‐language A used three consonants formed by the lips, that is, labial place: /b, v, w/, while those of mini‐language B used three consonants formed by the tongue tip, that is, coronal place: /d, z, l/). The long monophthongal English vowels /i, a, u, o, æ/ were used with the respective consonant sets to form the words of both languages, which were composed of three consonant‐vowel (CV) syllables (see Table 1) with different consonants and vowels in each of the three syllables (e.g., *bi‐va‐wo* for language A: lips, or *dæ‐zu‐la* for language B: tongue tip). Two hundred and 40 nonsense words were generated for each of the two mini‐languages.

**TABLE 1:** The consonant‐vowel (CV) syllables used to form the 3‐syllable words for mini‐languages A (labial: lips) and B (coronal: tongue tip).

		Vowel				
		i	a	o	u	æ
**Consonant language A (LIPS)**	**b**	bi	ba	bo	bu	bæ
	**v**	vi	va	vo	vu	væ
	**w**	wi	wa	wo	wu	wæ
**Consonant language B (TONGUE TIP)**	**d**	di	da	do	du	dæ
	**z**	zi	za	zo	zu	zæ
	**l**	li	la	lo	lu	læ

A 33‐year‐old female native speaker of Australian English was video‐ and audio‐recorded producing all 480 unique non‐words for the two mini‐languages in infant‐directed speech (IDS). She was born and raised in New South Wales, Australia. She reported no known speech or hearing disorders and presented no speech production problems. She gave informed consent to participate in the study and received $100 AU compensation for her participation.

The recording was conducted in a soundproof booth at the MARCS Institute for Brain, Behaviour and Development, Western Sydney University, Australia. The speaker was asked to produce the target non‐words in citation form as quickly but accurately as possible in blocks. Audio‐visual recordings were made via a Neumann microphone (https://en‐de.neumann.com) connected to a Sony camera and input to Adobe Premiere Pro (https://www.adobe.com/au/products/premiere.html) at 48 kHz 16‐bit audio, normalised at 70 dB SPL, and 25 fps video, 720 × 576 pixels. Video recordings captured the speaker's face from the neck up.

Audio‐only (A) and video‐only (V) stimulus words were separately extracted from the recordings. Audio non‐word tokens were segmented using Praat (Boersma and Weenink 2020), and then the best quality exemplar of each non‐word with similar duration was selected. Video tokens corresponding to the selected audio exemplars were segmented from the recording using Adobe Premiere and extracted without sound. Each video token began with a fully closed mouth just before the consonant and ended with an open mouth, as all words ended with a vowel. After extracting the videos, a black background was added to each token. The duration of all stimuli was adjusted to be within the target range of 750–1050 ms for audio and 1000–1300 ms for video by a trained phonetician using Adobe Audition (https://www.adobe.com/au/products/audition.html).

### 1.3 Procedure

The procedure was based on Kabdebon and Dehaene‐Lambertz's (2019) associative learning task, which employs a training phase and a test phase, but we used looking time (visual fixation) instead of EEG as the dependent measure. We programmed the procedure in Habit2 (Oakes et al. 2019).

**FIGURE 1:**
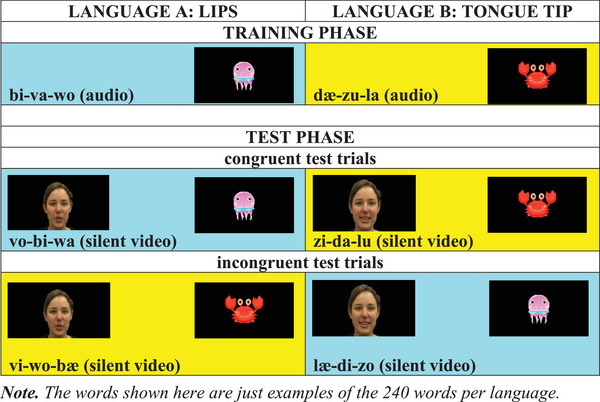
Examples of training and test phase trials. *Note*: The words shown here are just examples of the 240 words per language.

During the training phase, infants were exposed to two sets of word‐image pairings with audio‐only words. Mini‐language A (labial feature: lips) words were followed by a cartoon image of a jellyfish, and mini‐language B (coronal feature: tongue tip) words by a cartoon crab image. Infants completed 36 training trials divided into 3 blocks, always in the same order: 12 mini‐language A pairings, 12 mini‐language B pairings, and then 12 mixed mini‐language A and mini‐language B pairings (6 A pairings and 6 B pairings in pseudo‐random order). Test blocks followed immediately after training with no break. The test phase presented a modality shift: whereas infants had been presented with audio‐only words in the training phase, in the test phase they were presented with a video‐only face silently producing a word from the designated mini‐language. The test phase included 24 trials, of which 75% were congruent, that is, presented the trained word‐animal pairings (9 A‐word [lips—jellyfish] and 9 B‐word trials [tongue tip—crab]). The remaining 25% were incongruent, that is, presented word‐animal pairings (12.5%, 12.5%) that mismatched the training phase (3 A‐words [lips—crab]; 3 B‐words [tongue tip—jellyfish]) (see Figure 1). Test trials were presented in pseudo‐random order, such that 3‐trial sequences (triplets) of incongruent trials were separated by at least two triplets of congruent trials, with each 12‐trial block containing one incongruent (INC) triplet and three congruent (CON) triplets (e.g., CON‐A → CON‐B → INC‐A → CON‐B → CON‐A → INC‐B → CON‐A → CON‐B).

**FIGURE 2:**
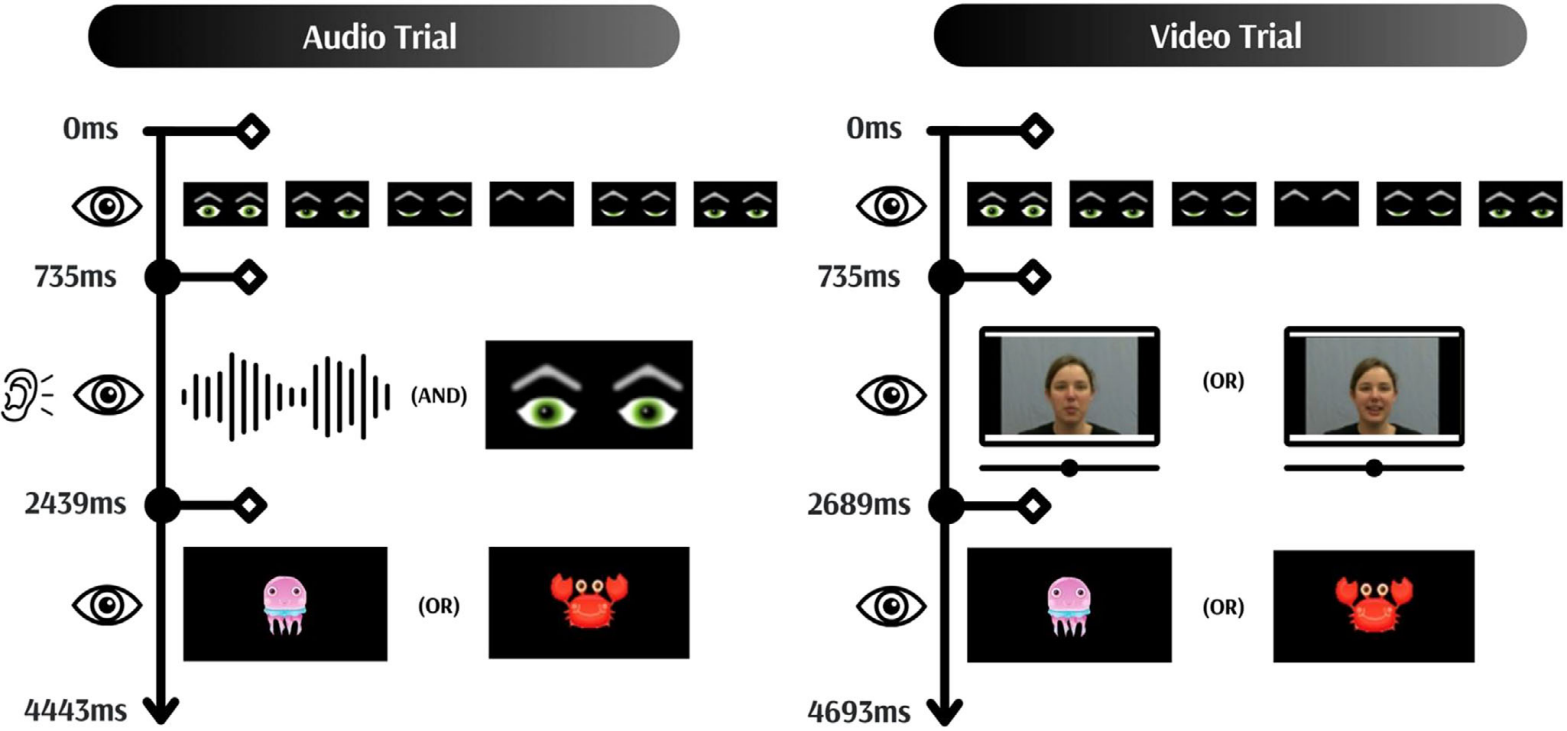
Illustration of sequence for audio trial and video trial.

In both phases, each trial began with a 735 ms period of silent blinking eyes to attract the infants’ attention to the screen. In the training phase, the audio word was presented along with static eyes on the screen for an average of 900 ms, followed by the static eyes alone for another 804 ms. In the test phase, the video of a silent face was presented for 1150 ms, followed by the static eyes alone for an average of 804 ms. After the presentation of the audio‐only or video‐only stimuli, a cartoon sea animal image appeared on the screen for 2004 ms, which was associated with the respective word type (training and congruent test trials: lips—jellyfish, tongue tip—crab; incongruent test trials: lips—crab, tongue tip—jellyfish). The duration of each audio‐only trial was 4.443 s, resulting in an overall training phase duration of 2.6 min. The duration of each video‐only trial was 4.693 s, resulting in an overall test phase duration of 1.8 min. The overall task lasted for 4.5 min. An illustration of the trial sequence can be seen in Figure 2. Further details can be found in Online Supporting Information.

Different subsets of the nonwords in each mini‐language were used for each task phase. Thus, infants never heard or saw the same word more than once in the study, and the words presented for each mini‐language were pseudorandomised across task phases and infants.

Participants were tested individually in a soundproof booth. While completing the task, infants sat on the parent's lap, facing a computer monitor approximately 135 cm away from the screen, and loudspeakers were located on either side of the monitor. The intensity of the stimuli was set to approximately 75 dB sound pressure level (SPL). Parents wore headphones and listened to music during the task to minimise the chance that they might inadvertently influence the infant's response to the stimuli.

### 1.4 Data Analysis

Infants’ looking times were video‐recorded by a hidden camera (Swann PRO‐640 infrared security camera) below the monitor during the experimental session and were coded offline using ELAN (2023). We transformed the raw‐looking times into proportions of looking time by dividing the looking time for each trial by the total duration of the trial. Although stimuli durations are within a specified range, slight variations still exist. By using proportions, we minimise any bias from these differences, ensuring that attention measures are not confounded by exact stimulus length. This allows for more accurate comparisons of attention across stimuli with different durations. This approach was applied to both the training trials and the test trials.

For the training phase analysis, the infants’ responses during the initial two blocks compared to the third block can reveal whether they have learned the word‐animal pairings for the two languages. This learning primarily occurs during the initial two single‐language blocks. If they have indeed acquired these associations, they would demonstrate familiarity during the subsequent mixed‐language block, resulting in reduced looking due to their recognition of the already‐learnt associations. For the test trial analysis, we included all trials in the test phase (18 congruent and 6 incongruent), rather than limiting the analysis to only the triplets preceding incongruent ones (6 congruent and 6 incongruent), as done by Kabdebon and Dehaene‐Lambertz (2019). Additional analyses following their method can be found in the Supporting Information.

The statistical analyses for each phase of the study were performed with binomial generalised linear mixed‐effects (GLME) models in *R* (R Team), using the *glm* function from the package *lme4* (Bates et al. 2014). The *p* values for the fixed‐effects factors were computed using Satterthwaite's method, and the *Anova* function from package *car* (Fox and Weisberg 2018) was used to perform an analysis of variance, which assesses the significance of the fixed effects in the models. The dependent variable was the proportion of looking time per trial. We first conducted the analysis of the looking times during the training phase. This initial step allowed us to assess infants’ attention and interest in the stimuli during the learning phase prior to the introduction of incongruent trials in the test phase. For the training phase analysis, the fixed effect was block (Block 1: 12 language A pairings; Block 2: 12 language B pairings; Block 3: 12 mixed A and B pairings). Participants were the random effect. Here we included all trials, and they were all congruent. For the subsequent test phase analysis, the fixed effect was condition (congruent, incongruent). Here, again we included all trials. Participants and trials were random effects. Contrast coding was applied to the block for the training phase analysis (Block 1 vs. Block 2 coded as Block 1 = −1, Block 2 = 1; and for the Block 2 vs. Block 3 analysis: Block 2 = −1, Block 3 = 1) and condition for the test phase analysis (congruent = −1, incongruent = 1) using the generalised inverse method to enable comparisons between different levels.

## 2 Results

### 2.1 Training Phase

First, we conducted an analysis of the looking time proportion during the training phase to evaluate changes in proportion of looking time across blocks as an index of learning (1 [12 language A pairings], 2 [12 language B pairings], and 3 [12 mixed A and B pairings]). The model showed a better fit to the data, as indicated by the lower AIC (434.54) and BIC (454.98) values, when compared to both the null model and the model that incorporates trial order as an additional random effect.

**FIGURE 3:**
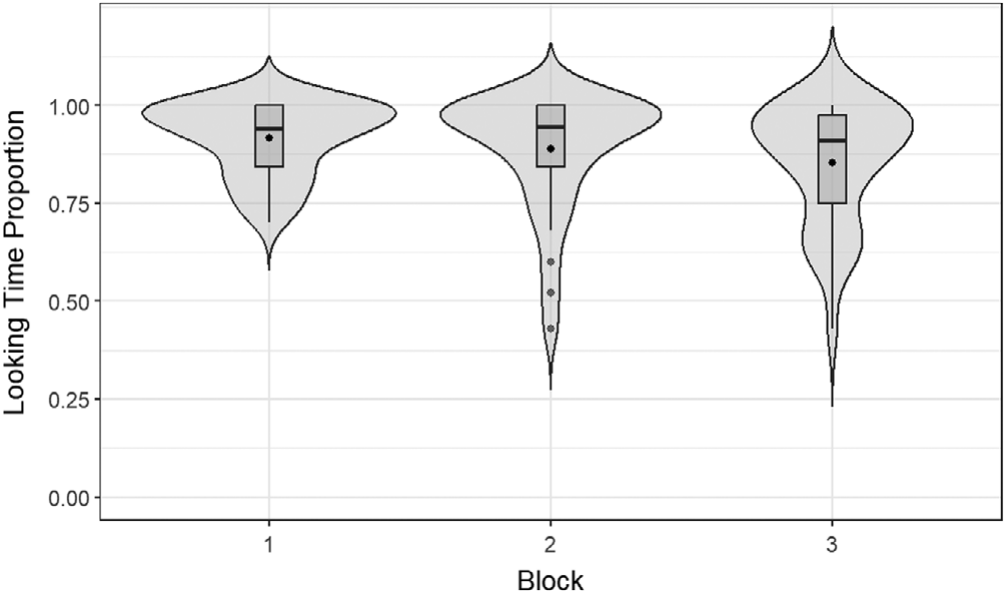
Looking time proportion for each block in the training phase. *Note*: Block 1: language A pairings; Block 2: language B pairings; Block 3: mixed A and B pairings. Boxplots in each violin plot show the quartiles and potential outliers for each block. The dot in the middle of each rectangle within each violin shows the mean and the line shows the median for that block.

We observed a significant main effect of block (*χ* ^2^ = 6.6868, df = 2, *p* = 0.035). Specifically, Block 3 exhibited a negative effect (*β* = −0.90, SE = 0.36, 95% CI [−1.62, −0.19], z = −2.475, *p* = 0.013), indicating significantly lower looking time proportions in this block than in Blocks 1 and 2 (see Figure 3). This implies that the infants had learned the word‐animal associations during the first two blocks and recognised the trained pairings in the final mixed block.

## 3 Test Phase

The model fit statistics indicate that the model with participant as a random effect provides a better fit to the data (AIC = 700.21, BIC = 719.03) in comparison to the null model and the model with participant and trial as random effects.

**FIGURE 4:**
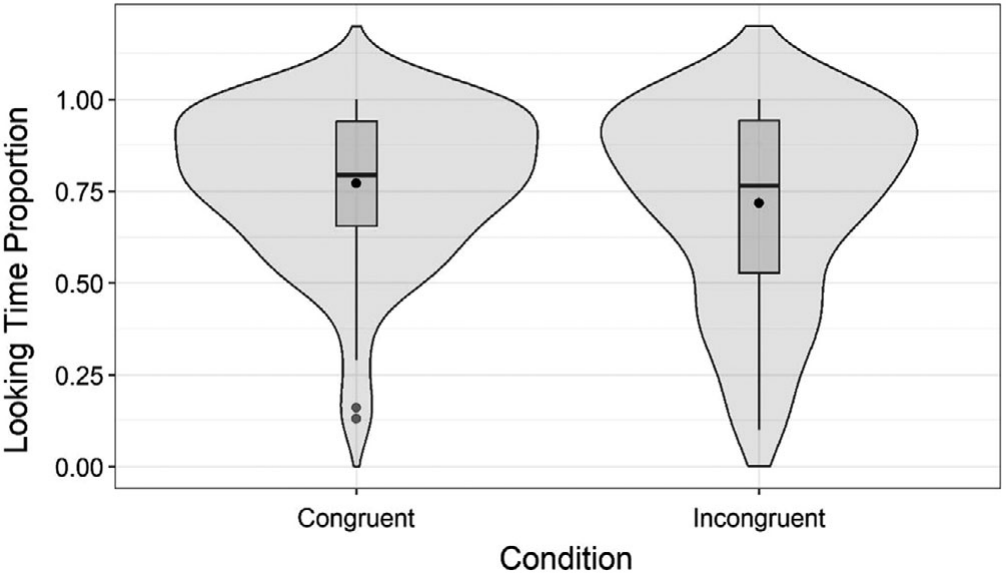
Looking time proportion for the congruent and incongruent conditions in the test phase. *Note*: The violin plots display the distribution of the data for each condition (congruent, incongruent) along the *y*‐axis (looking time proportion). The width and shape of the violin plot show the density of the data at different values. Boxplots in each violin plot show the quartiles for each condition. The dot in the middle of each rectangle within each violin shows the mean and the line shows the median for that condition.

There was a significant main effect of condition (*χ* ^2^ = 7.0538, df = 1, *p* = 0.007), indicating that looking time was lower in the incongruent trials than in the congruent trials (*β *= −0.68, SE = 0.2561, 95% CI [−1.18, −0.18], *z *= −2.656, *p* = 0.007). See Figure 4 for these results.

## 4 Discussion

In this study, we investigated whether young infants (< 6 months) could detect articulatory information shared across multiple consonants of the words in each of two mini‐languages, allowing them to differentiate that the lexicons of the languages contrasted solely by the phonological feature of place of articulation: labial (lips) versus coronal (tongue tip). The consonants of all words in the two mini‐languages used contrasting speech articulators: lips (Language A) versus tongue tip (Language B). Generalisation was assured by using multiple consonants sharing an articulatory feature in each word and by never using the same word twice within or between phases of the experiment. A cross‐modal design was implemented to examine whether their learning of that place of articulation feature is amodal, that is, phonologically abstract, rather than being restricted to just the training modality (audio), which would not unequivocally indicate the abstract nature of the shared phonological feature.

Our findings in the training phase with audio‐only words revealed a significant main effect of block. The infants’ significantly shorter looking time during the third block (mixed trials of mini‐languages A and B) than during the initial two single‐language blocks (A then B) suggests that they had abstracted the articulatory feature for each mini‐language during the first two blocks. That is, their reduced looking during the third, mixed‐language block implies they were already familiar with the two word‐animal associations, which they had presumably learned during the initial two single‐language training blocks. Similarly, findings from Plunkett, Hu, and Cohen (2008) indicated a reduction in infants’ looking time during familiarisation across multiple experiments, suggesting habituation. Those authors interpreted this as indicating that infants retained memory of the standard stimulus over time. Thus, our interpretation aligns with previous research in infant studies, in which decreased looking time to previously presented stimuli is interpreted as reflecting encoding (extraction and retention in memory) of the core properties of the stimuli (see also Fernald 1985; Houston‐Price and Nakai 2004; Hunnius and Geuze 2004; Rose et al. 2002).

In the test phase, we found a significant main effect of condition. There was a significantly lower looking time when the condition was incongruent than when it was congruent, indicating that young infants had abstracted the phonological feature for the articulatory difference between two artificial languages (lips/labial vs. tongue‐tip/coronal consonants) during the training phase, and in the subsequent test phase they preferred the familiar word‐animal pairings they had learned during training. Importantly, infants maintained this abstract phonological learning across the speech modality change from audio‐only (A) in training to video‐only (V) at test. The test results cannot be attributed to any other dissimilarities between the nonwords of mini‐language A and mini‐language B, as infants were equally exposed to both mini‐languages in the training phase.

Based on our findings, we can establish several connections to the infant studies introduced earlier. Our research not only corroborates the cross‐modal results of Pons et al. (2009) and Best et al. (2010) but also introduces unique dimensions to the existing body of literature. While sharing cross‐modal methods and a common focus on place of articulation distinctions with both aforementioned studies, our study stands out by employing a more exacting test of abstraction of the contrasting place features. Instead of solely investigating a single consonant contrast, such as [ba] versus [va] (bilabial vs. labio‐dental) as in Pons et al. (2009) or [pa] versus [ta] as in Best et al. (2010), our study incorporated multiple consonants within each place of articulation and never presented the same word twice, thereby providing infants with the more abstract challenges of extracting the phonological feature shared across the consonants of each mini‐language and also the feature difference between the languages. This approach allows us to draw inferences about phonological abstraction of place of articulation rather than only about discrimination of a specific consonant contrast (or specific words or syllables).

The findings suggest that infants rapidly acquire abstract phonological knowledge during the training phase, and they maintain this abstraction across a modality shift from audio during training to video in the test phase. This is a significant departure from the conventional assumption that infants first need to attune to perceptual distinctions in their native language before they can abstract phonological features. Our results instead align with the results of the Choi, Broersma, and Cutler (2017) study in which, compared to native Dutch adults, adults adopted from Korea to the Netherlands at 3 to 5 months, with no further exposure to their birth language, learned a Korean consonant feature contrast more quickly and abstracted that feature to a new place of articulation. The authors inferred from those results that phonological feature abstraction can occur before 6 months, that is, prior to perceptual attunement, and our results here support that inference. Our findings provide direct evidence for such early abstraction abilities, indicating that infants under 6 months are capable of learning a new phonetic contrast and generalising this learning simultaneously in two ways: (i) via an abstract phonological representation to a new place of articulation and (ii) via an amodal representation to new tokens presented in a different modality. These two findings support the hypothesis that infants have both phonologically abstract and amodal representations, at least for this phonological feature contrast by 4–6 months, prior to the emergence of perceptual attunement around 6–10 months.

This insight, in turn, underscores the need for more flexible and dynamic theoretical models that can accommodate the possibility of phonological feature abstraction preceding perceptual attunement or even the possibility of parallel development of the two skills. It encourages a re‐examination of theories such as Jusczyk's WRAPSA (1993), which posits that perceptual attunement precedes phonological feature abstraction, and the PRIMIR framework (Werker and Curtin 2005), which emphasises the importance of word learning as a prerequisite for phonological abstraction. Our findings also have significant implications for the PAM (Best 1993, 1994). PAM posits that the foundation of speech perception, including both perceptual attunement and phonological feature abstraction, is based on amodal articulatory information. The successful cross‐modal transfer we observed here is consistent with this PAM premise and extends it to young infants’ ability to abstract and generalise a phonological feature contrast on the basis of amodal articulatory information.

As anticipated, our study's results revealed that infants paid more attention and had longer looking times during congruent test trials compared to incongruent ones. This preference aligns with previous findings on infants’ familiarity and novelty preferences. In a detailed review of conditions under which there are preferences for familiar and for novel stimuli, Burnham and Dodd (1998) discussed six factors that led to familiarity preferences. Three of these are relevant here; they are familiarity preferences (a) for linguistic stimuli, (b) in younger infants (in the range tested here), and (c) when the task requires the infants to use an amodal code. In our study, our stimuli are linguistic, involving words from each mini‐language that differ in their consonants (labial and coronal). The infants are 4–6 months old, consistent with the preference observed in younger infants. Additionally, the task required infants to use an amodal code by abstracting articulatory information from speech across different modalities, specifically from audio‐only to video‐only (silent talking face) presentations of spoken words. In the congruent trials, new words from each mini‐language were paired with the same animal image encountered during the training phase, providing a familiar stimulus context. This combination of familiar linguistic stimuli and the requirement to use an amodal code likely contributed to the heightened attention and preference observed for the congruent test trials.

Our findings are also consistent with those of Kabdebon and Dehaene‐Lambertz (2019) in demonstrating the ability of abstract linguistic processing of infants under 6 months. In their research, preverbal infants exhibited a remarkable ability to distinguish between two different syllable structures of nonwords and associate them with different visual images of animals. What is particularly striking is that the infants in their study displayed the capacity to generalise this knowledge to novel trials, even when the order of presentation was reversed, with the animal image preceding rather than following the auditory word. Our study serves as a substantive conceptual and methodological extension of their study, with a specific focus on phonological feature abstraction across different sensory modalities. While Kabdebon and Dehaene‐Lambertz (2019) employed high‐density EEG recordings to uncover infants' formation of expectations and surprise in response to violations of the trained word structure‐animal associations, our study took a different approach. We adapted their mini‐language association task to a behavioural paradigm that used a looking time measure, with a specific focus on phonological feature abstraction and infants’ differentiation between languages based on the labial (lips) versus coronal (tongue tip) place of articulation feature contrast. Employing a cross‐modal design, we provided evidence of phonological feature abstraction, thus supporting the idea that infants possess an abstract amodal representation of these phonological contrasts in consonant place of articulation even before reaching 6 months of age. Although our study and the Kabdebon and Dehaene–Lambertz study diverged in their areas of emphasis and measures of learning, they converge in their evidence for early linguistic abstraction processes in infants.

Two alternative explanations^1^ of our results should be considered, however. First, it is possible that the infants may have learned the specific sets of three consonants used in each mini‐language without necessarily abstracting a common place of articulation (POA) feature for either language. During the test phase, the infants could have recognised whether the consonant set was followed by the trained animal associated with that set or by an untrained animal associated with the other set, without relying on the abstraction of a POA feature. To distinguish between our POA feature abstraction account and this alternative, a future study could train infants on two mini‐languages for which neither consonant set shares a common POA, for example, /b, s, l/ versus /p, z, r/. If the infants perform similarly to the present study even with the non‐shared feature mini‐languages, the alternative account would gain support, suggesting that the infants in the current study also may have simply learned two sets of three consonants. But if the infants fail to show a preference in the test phase under the non‐shared features mini‐languages, the difference from our current findings would support our articulatory feature abstraction account.

Another possibility is that infants might have already learned, in their everyday interactions before entering this study, the associations between the auditory and visual properties of the consonants used in each mini‐language, which would mean they did not need to abstract amodal articulatory information to perform as they did on the video test trials following audio training. To distinguish between our hypothesis and this ‘known audiovisual properties’ account, a future study could use sets of unfamiliar, non‐native consonants that the infants have not previously encountered but that are still grouped by labial versus coronal place of articulation (POA). For instance, a non‐native coronal set could include the Zulu voiced lateral fricative [ɮ] (instead of [z]), the Malayalam dental nasal [n̪] (instead of [n]), and the Russian palatalised lateral glide [ʎ] (instead of [l]). For the labial set, consonants might include the Danish labiodental approximant [ʋ] (instead of [w]), the Fijian bilabial fricative [β] (instead of [v]), and the Kukuya labiodental nasal [ɱ] (instead of [b]). If the infants fail to generalise in the video test phase, the difference in results would support this alternative hypothesis. However, if they do show the same preference as in the present study, that would indicate confirmation of our articulatory feature abstraction account. While such a study would be informative, we note that other recent findings already support the idea that infants under 6 months can form phonological abstractions, specifically for the initial consonant of varying words and for differing syllabic structures (Mersad et al. 2021; Santolin et al. 2024).

While our study established the presence of amodal phonological feature abstraction at 4–6 months, particularly in a cross‐modal context, further investigations are necessary to examine abstraction within the audio‐only and video‐only modalities. Within an audio‐only study, in which stimuli remain within the same modality for both training and test phases, we anticipate a novelty effect for the incongruent trials, that is, greater looking time towards the incongruent stimuli (incongruent > congruent looking). This prediction is grounded in the assumption that infants will prefer moderate novelty from the training phase, which would result in a preference for the incongruent trials as novel, moderated by the familiarity of the audio modality. Future research could explore these and other issues, such as whether young infants can generalise to new consonants at the trained place of articulation and whether they learn artificial languages better if the consonants share an articulatory feature than if they do not (phonologically arbitrary consonant sets). Addressing these additional questions will provide a comprehensive understanding of place of articulation abstraction and its modality‐specific effects, enhancing our knowledge of infants’ phonological processing abilities.

Recent findings by Di Liberto, Attaheri, Cantisani et al. (2023) may seem to differ from ours with respect to onset age. Their longitudinal study presented sung nursery rhymes to 4‐, 7‐, and 10‐month‐old infants and found EEG tracking of phonetic features only by 7 months, not at 4 months, implying that the brain response to phonetic features in sung nursery rhymes may appear only after perceptual attunement to vowels emerges. While the authors argued that their paradigm is more ecologically valid for early language development investigations than are experimental procedures such as ours, sung nursery rhymes also differ ecologically from natural spontaneous speech interactions with infants and do not straightforwardly lend themselves to assessment of infant's learning of the role those features play in the lexicon of a language. Those differences from our controlled experimental paradigm, which used natural spoken (not sung) words and did assess for learning of two lexica that differ in specific abstract phonological features, may well account for the age difference in findings. Thus, neither our study nor the de Liberto study fully capture the richness and complexity of infants’ *linguistic* experiences in real‐life spoken interactions. Nevertheless, both studies contribute valuable insights into the early emergence of phonological feature abilities in infancy. Future research could aim to bridge the gap between controlled laboratory paradigms and ecologically valid approaches to further elucidate the mechanisms underlying language acquisition in early infancy.

While our suggested future studies would further flesh out the nature of phonological abstraction on the basis of our results, we can conclude that infants under 6 months can not only learn two mini‐languages that differ in the place of articulation feature of their consonants but also can generalise that feature across modalities. This supports the hypothesis that infants develop both phonologically abstract and amodal representations of phonological features by 4–6 months, before perceptual attunement occurs.

## Ethics

The study was conducted at the MARCS Institute for Brain, Behaviour and Development under Western Sydney University Human Research Ethics Committee approval in accordance with the Code of Ethics of the World Medical Association (Declaration of Helsinki).

## Conflicts of Interest

The authors declare no conflicts of interest.

## Supporting information



Supporting Information

## Data Availability

The data that support the findings of this study are available from the corresponding author upon reasonable request.

## Endnotes

1 We thank an anonymous reviewer for these suggestions.
