# Supplementary material for: Phonological Feature Abstraction Before 6 Months: Amodal Recognition of Place of Articulation Across Multiple Consonants
Source: Dev Sci. 2025 Jan 14;28(2):e13605. doi: 10.1111/desc.13605 (PMC11733024; doi:10.1111/desc.13605)
Supplement: Supplementary file 1 — Supporting Information [file DESC-28-e13605-s001.docx]

**ONLINE SUPPLEMENTARY MATERIALS:** TRIAL STRUCTURE

**AUDIO Trial structure**: each trial has 4 steps; repeat trials until the specified block is complete.

**total trial duration = 4443 milliseconds (ms) for audio trials.**

1. (duration: 735 ms) **Blinking eyes animation**: static images are presented in sequence as indicated below for the indicated ms durations. They are presented in silence (no audio).

Repeat sequence twice (note different timing for the first image [openXX.jpg] on the second time

through), then the [openXX.jpg] image remains on screen through steps 2-3 (below):

1^st^ time: 133 ms

2^nd^ time: 72 ms 33 ms 66 ms 66 ms 66 ms 33 ms


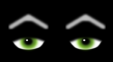

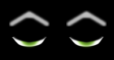

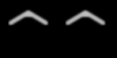

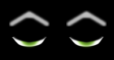

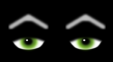

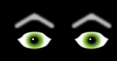
**Screen**:

e.g., [openGREEN.jpg] [partialGREEN.jpg] [midGREEN.jpg][closedGREEN.jpg] [midGREEN.jpg][partialGREEN.jpg]

1. (mean of 900 ms) **Audio word presentation**: the designated static open eyes image (e.g., [opeGREEN.jpg]) comes up on the screen at the end of the second blink sequence, and remains up throughout a single audio word presentation and silent step 3 (below). Audio words are each 900 ms in duration on average (750-1050 ms range). The program selects a word randomly and without replacement from the designated word type list/folder for the specified phase and block of the procedure, and plays the selected word out.


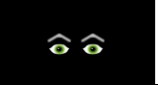


+ bi-va-wo (audio)

e.g., [openGREEN.jpg] e.g., [Aa-exp87.wav]

*cumulative:* 1704 ms

1.
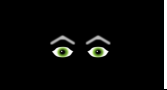
(mean of 804 ms) **Silent static eyes**: the designated static open eyes image (e.g., [openGREEN.jpg]) remains on the screen for an average of 804 ms; this phase is silent (no audio)

e.g., [openGREEN.jpg]

*cumulative:* 2439 ms

1. (2004 ms)  **Image associated with the designated word type**: silent presentation (no audio) of the designated image for the word type (A or B) assigned to the specified phase and block of the experiment.


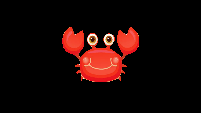


e.g., [Crab.jpg]

*cumulative:* 4443 ms (i.e., total trial duration)


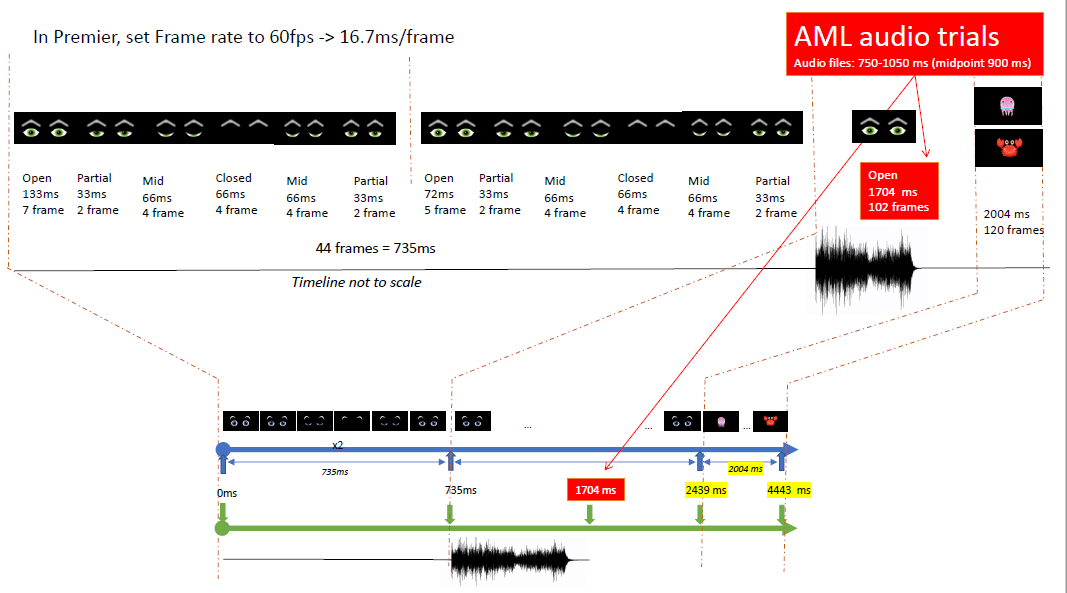


**VIDEO Trial structure**: each trial has 4 steps; repeat trials until the specified block is complete

**total trial duration = 4693 milliseconds (ms) for video trials.**

1. (duration: 735 ms) **Blinking eyes animation**: static images are presented in sequence as indicated below for the indicated ms durations. They are presented in silence (no audio).

Repeat sequence twice (note different timing for the first image [openXX.jpg] on the second time through), then the [openXX.jpg] image remains on screen through steps 2-3 (below):

1^st^ time: 133 ms


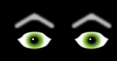

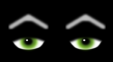

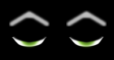

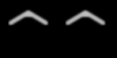

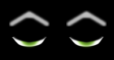

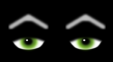
2^nd^ time: 72 ms 33 ms 66 ms 66 ms 66 ms 33 ms

**Screen**:

e.g., [openGREEN.jpg] [partialGREEN.jpg][midGREEN.jpg] [closedGREEN.jpg] [midGREEN.jpg] [partialGREEN.jpg]

1.
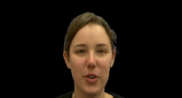
(mean of 1150 ms) **Silent video word presentation**: the designated static open eyes image (e.g., [opeGREEN.jpg]) stays on the screen at the end of the second blink sequence, and remains up throughout a single video word presentation which is superimposed on the static eyes (video hides eyes while playing), and silent step 3 (below). Video words are each 1150 ms in duration (1000-1300 ms range). The program plays out the word from the designated word type list/folder for the specified phase and block of the procedure,

+

e.g., [openGREEN.jpg] e.g., [Aa-exp87.mp4]

(hidden behind the video word)

1. (mean of 804 ms) **Silent static eyes**: the designated static open eyes image (e.g., [openGREEN.jpg]) remains on the screen for 804 ms; this phase is silent (no audio)


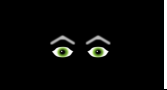


e.g., [openGREEN.jpg]

*cumulative:* 1954 ms

1. (2004 ms) **Image associated with the designated word type**: silent presentation (no audio) of the designated image for the word type (A or B) assigned to the specified phase and block of the experiment.


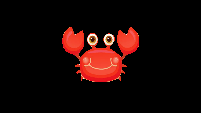


e.g., [Crab.jpg]

*cumulative:* 4693 ms (i.e., total trial duration)

**
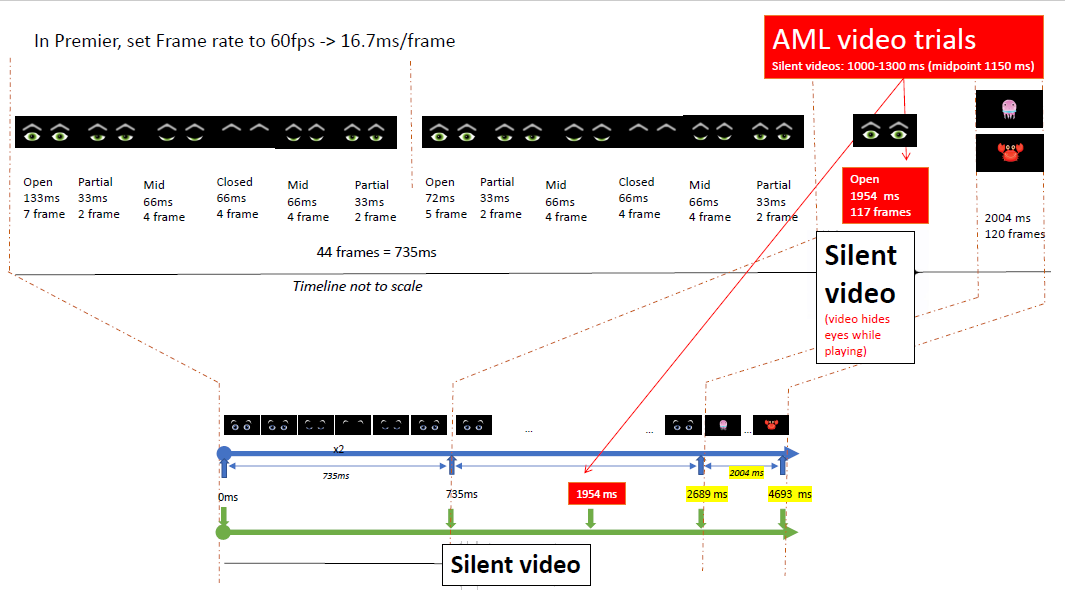
**

**ONLINE SUPPLEMENTARY MATERIALS:** Analysis with Only the Congruent Triplets Preceding Incongruent Ones

We reanalysed the data by including only the triplets preceding incongruent ones (6 congruent and 6 incongruent) in the test phase, as was done by Kabdebon and Dehaene-Lambertz (2019). We fitted a GLM model to predict looking time proportion with condition and block as the predictors, with participant as a random effect. The model fit statistics indicate that the model provides a better fit to the data (AIC = 364.98, BIC = 385.03), in comparison to the null model, the model that only includes condition as the fixed effect thus excluding block as a fixed effect, and the model that includes block as a random effect. There was a significant main effect of condition (*χ²* = 6.0632, df = 1, *p* = 0.013), indicating that looking time was less in the incongruent than the congruent trials (*β* = -0.67, *SE* = 0.2352, 95% CI [-1.25, -0.09], *z* = -2.249, *p* = 0.024). A significant main effect of block was also found (*χ²* = 15.5936, df = 1, *p* < 0.001). Infants exhibited longer looking time in block 1 than block 2 (*β* = -1.16, *SE* = 0.3012, 95% CI [-1.75, -0.57], *z* = -3.837, *p* < 0.001). However, the condition x block interaction was not significant (*χ²* = 0.1661, df = 1, *p* = 0.683).

**ONLINE SUPPLEMENTARY MATERIALS:** Analysis with Only the Incongruent Triplets Preceding Congruent Ones

We fitted another model which includes the congruent triples following (instead of preceding) the incongruent ones (6 incongruent and 6 congruent) to ensure that the decline in looking time for incongruent trials in the Kabdebon and Dehaene-Lambertz approach above is not simply because they always follow congruent trials. We fitted a GLM model to predict looking time proportion with condition and block as the predictors, with participant as a random effect. The model fit statistics indicate that this model provides a better fit to the data (AIC = 346.3, BIC = 366.3) compared to the null model, the model that only includes condition as the fixed effect thus excluding block as a fixed effect, and the model that includes block as a random effect. There was a main effect of condition (*χ²* = 2.8294, df = 1, *p* = 0.040), indicating that looking time was lower in the incongruent trials than in the congruent trials (*β* = -0.67, *SE* = 0.3282, 95% CI [-1.32, -0.03], *z* = -2.048, *p* = 0.040).
